# The identification of co-expressed gene modules in *Streptococcus pneumonia* from colonization to infection to predict novel potential virulence genes

**DOI:** 10.1186/s12866-020-02059-0

**Published:** 2020-12-17

**Authors:** Sadegh Azimzadeh Jamalkandi, Morteza Kouhsar, Jafar Salimian, Ali Ahmadi

**Affiliations:** 1 Chemical Injuries Research Center, Systems Biology and Poisonings Institute, Baqiyatallah University of Medical Sciences, Tehran, Iran; 2 Laboratory of Systems Biology and Bioinformatics (LBB), Institute of Biochemistry and Biophysics (IBB), University of Tehran, Tehran, Iran; 3 Molecular Biology Research Center, Systems Biology and Poisonings Institute, Baqiyatallah University of Medical Sciences, Tehran, Iran

**Keywords:** *Streptococcus pneumonia*, Transcriptome analysis, SDP algorithm, Systems biology

## Abstract

**Background:**

*Streptococcus pneumonia* (pneumococcus) is a human bacterial pathogen causing a range of mild to severe infections. The complicated transcriptome patterns of pneumococci during the colonization to infection process in the human body are usually determined by measuring the expression of essential virulence genes and the comparison of pathogenic with non-pathogenic bacteria through microarray analyses. As systems biology studies have demonstrated, critical co-expressing modules and genes may serve as key players in biological processes. Generally, Sample Progression Discovery (SPD) is a computational approach traditionally used to decipher biological progression trends and their corresponding gene modules (clusters) in different clinical samples underlying a microarray dataset. The present study aimed to investigate the bacterial gene expression pattern from colonization to severe infection periods (specimens isolated from the nasopharynx, lung, blood, and brain) to find new genes/gene modules associated with the infection progression. This strategy may lead to finding novel gene candidates for vaccines or drug design.

**Results:**

The results included essential genes whose expression patterns varied in different bacterial conditions and have not been investigated in similar studies.

**Conclusions:**

In conclusion, the SPD algorithm, along with differentially expressed genes detection, can offer new ways of discovering new therapeutic or vaccine targeted gene products.

**Supplementary Information:**

The online version contains supplementary material available at 10.1186/s12866-020-02059-0.

## Background

*Streptococcus pneumonia* (pneumococci) is a common bacterial pathogen in children, immunocompromised individuals, and the elderly. It infects the upper respiratory tract (especially nasopharynx) of 27–65% of children and 10% of adults. Pneumococci can cause severe infections in susceptible hosts through a highly flexible gene expression capacity, allowing it to move from the nasopharynx and adapt to highly sterile body sites, including lung, blood, and brain. It causes a wide range of disorders, from otitis media and sinusitis to severe infections, such as bacteremia, pneumonia, and meningitis [1]. Hence, despite available pneumococcal treatments and effective vaccines, pneumococci is one of the 12 highly invasive pathogens causing more deaths than any other infectious diseases in the world [2]. A small pneumococcal genome size (3000–5000 genes) confirms that transcriptional events play a critical role in an adaptive and smart behavior [3]. Accordingly, many studies have investigated the pathogenicity behavior of the pathogen by measuring, through microarray experiments, the expression of essential virulence genes and comparing it with that of non-pathogenic bacteria in different niches during colonization and invasion [4]. As systems biology studies have demonstrated, critical co-expressing modules and genes may serve as the key player in biological processes. Generally, Sample Progression Discovery (SPD) is a computational approach traditionally used to decipher biological progression trends and their corresponding gene module clusters in different clinical samples underlying a microarray dataset. This approach is used in progression-based diseases, including cancer, chronic pulmonary obstructive disease (COPD), and basic cellular processes, including cell differentiation [5]. The SPD framework tries to cluster genes into modules of co-expressed genes, construct modules’ minimum spanning tree (MST), select modules corresponding to common MSTs, and, according to all genes of all selected modules, reconstruct a general MST [6]. Because some essential virulence genes may not necessarily be differentially expressed genes (DEGs), we aimed to use SPD to define if some critical niche dependent-, co-expressed modules, and genes are necessary for bacterial translocation through different host niches during the pathogenicity adventure of the pathogen. In the present study, we wonder if some genes are ignored during the DEGs detection approach while they may potentiate to serve as key candidate regulators in the pathology trend of pneumococcal diseases. We aimed to analyze pneumococcal’s gene expression behavior in both colonization and invasion states by focusing on two microarray datasets of pneumococcus derived from the nasopharynx, lung, blood, and brain of mice. The data were analyzed through a machine-learning algorithm to detect those genes related to the infection progression from the nasopharynx to the lung, blood, and brain. We finally found some key expression modules and genes that could distinguish precisely between different clinical samples.

## Results

### Co-expressed modules detected by SPD

According to the aim of the present study, the two extracted datasets were pooled to analyze with limma. Each spot file contained 3297 unique probes, of which 943 included control probes that were filtered out in the pre-processing step. Then, the gene expression matrix was reconstructed as the input of SPD. As a result of the SPD algorithm, many co-expressed modules were obtained based on time series data in each group. After investigating all modules and their MST, modules that significantly separated the samples based on their gene expression patterns and source tissues (including nasopharynx, lung, blood, and brain) were selected to further analysis (Table 1, S1 File). Although the enrichment analysis and literature mining could not find any meaningful information about many of the modules due to limited genetic annotations and enrichment tools on *Streptococcus pneumonia*, some critical modules were identified in each group, including genes involved in essential cellular processes. Among these, top modules are analyzed in the following section (S1 Table).

**Table 1:** The number of modules extracted from the data by the SPD algorithm

Sample source	No. of all modules	No. of selected modules	No. of top modules (No. of each module’s genes)
Nasopharynx and lung	182	30	2 (13, 10)
Lung and blood	160	10	2 (7, 9)
Blood and brain	138	13	2 (3, 14)
Nasopharynx, lung, and blood	179	15	2 (5, 8)
Lung, blood, and brain	160	12	1 (3)
Nasopharynx, lung, blood, and brain	169	18	2 (4, 3)

#### Nasopharynx-lung progression

**Fig. 1:**
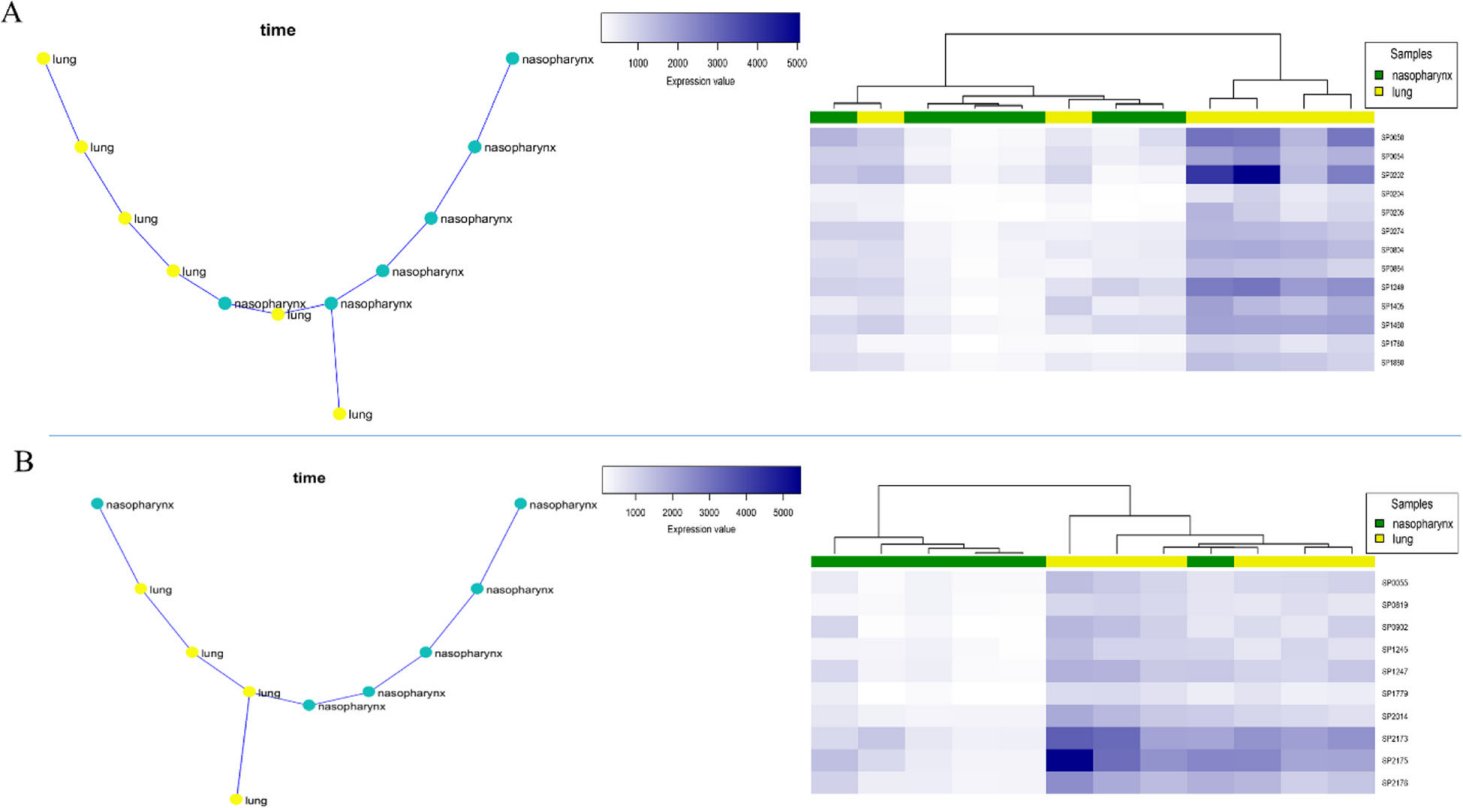
**a** The co-expressed module 14 represents the infection progression in the nasopharynx to lung (Left: MST, Right: hierarchical clustering based on gene expression data). **b** The co-expressed module 71 represents the infection progression in the nasopharynx to lung (Left: MST, Right: hierarchical clustering based on gene expression data)

Regarding the nasopharynx-lung expression data, a total of 182 modules were detected by SPD, two of which (modules 14 and 71) were selected as the best results based on their MST, representing the invasion of bacteria from the nasopharynx to the lung (Fig. 1). As shown in Fig. 1, based on MST and hierarchical clustering, results showed that these modules’ gene expression pattern is significantly different between lung and nasopharynx. The genes involved in module 14 are mostly enriched to the “purine metabolism” pathway and “*‘de novo*’ inosine monophosphate (IMP) biosynthetic” process (S2 Table). These pathways and processes are related to biofilm formation [7, 8]. In module 71, three genes (SP_2173, SP_2175, and SP_2176) are involved in host immune system defensive mechanisms against infections, including the “Cationic antimicrobial peptide (CAMP) resistance” pathway that contains six genes [9, 10]. Another pathway in module 71 is the “Two-component regulatory system,” a pathway that regulates the expression of pneumococcal surface antigen A protein and consequently controls the virulence of bacteria and its resistance oxidative stress [11, 12]. The two-component system is also related to Cellobiose Metabolism and the interaction of the bacteria with its environment [13, 14].

Literature mining demonstrated that the module 14 genes are very important in pneumococcal infection. For instance, SP_0050 (purH), SP_0202 (nrdD), SP_0054 (purK) and SP_0205 (nrdG) are early pneumococcal response genes in human lung epithelial cells [15]. Iron starvation condition up-regulates the expression of nrdD, SP_0204, and SP_0205 genes, whereas it down-regulates purK, SP_1405, and SP_1460 genes [16]. In addition, SP_1780, SP_1405 (spxA), and SP_0274 (polC) are reported to be pneumococcal essential genes for pulmonary infection [17].

#### Lung-blood progression

**Fig. 2:**
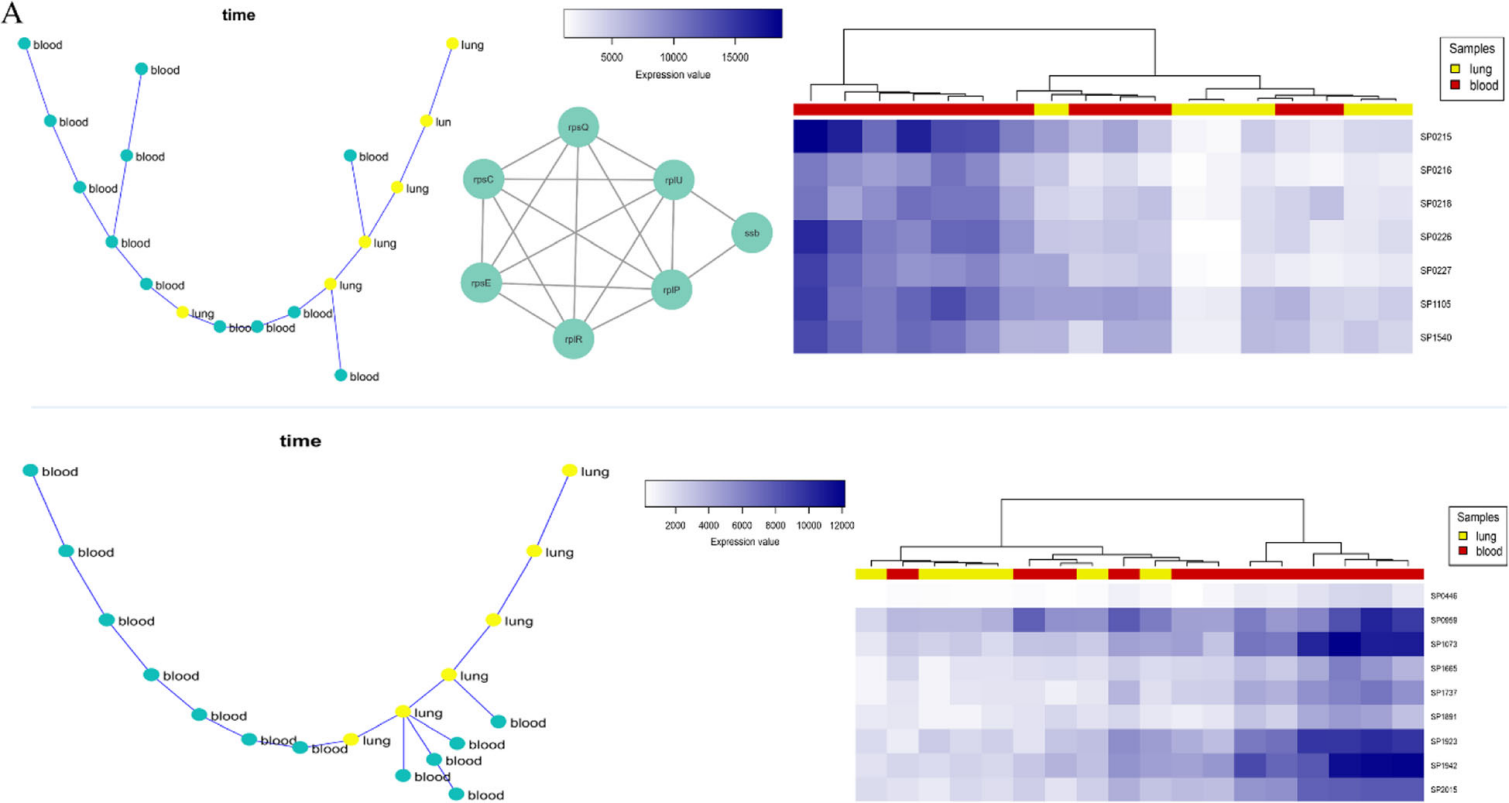
**a** The co-expressed module 22 represents the infection progression in lung to blood (Left to right: MST, protein interaction network, and hierarchical clustering based on gene expression data). **b** The co-expressed module 101 represents the infection progression in lung to blood (Left: MST, Right: hierarchical clustering based on gene expression data)

Applying the SPD algorithm on lung-blood expression data detected 160 co-expressed modules, two of which (modules 22 and 101) were selected for further analysis. As shown in Fig. 2, these modules’ expression pattern was significantly different between lung and blood samples. All proteins in module 22 interact physically with each other based on bacterial interactome (Fig. 2a). The genes in this module significantly enriched the “Ribosome” pathway and some significant processes, such as “nitrogen compound metabolic process,” “primary metabolic process,” and “response to stimulus” (S2 Table). The nitrogen compound metabolic process and primary metabolic process are dysregulated in copper resistance *Streptococcus pneumonia* [18]. No enrichment result was found for module 101, though some critical previously reported genes were present in this module. For example, Giefing et al. [19] introduced the SP_1923 and SP_1891 genes as vaccine candidates.

#### Blood-brain progression

**Fig. 3:**
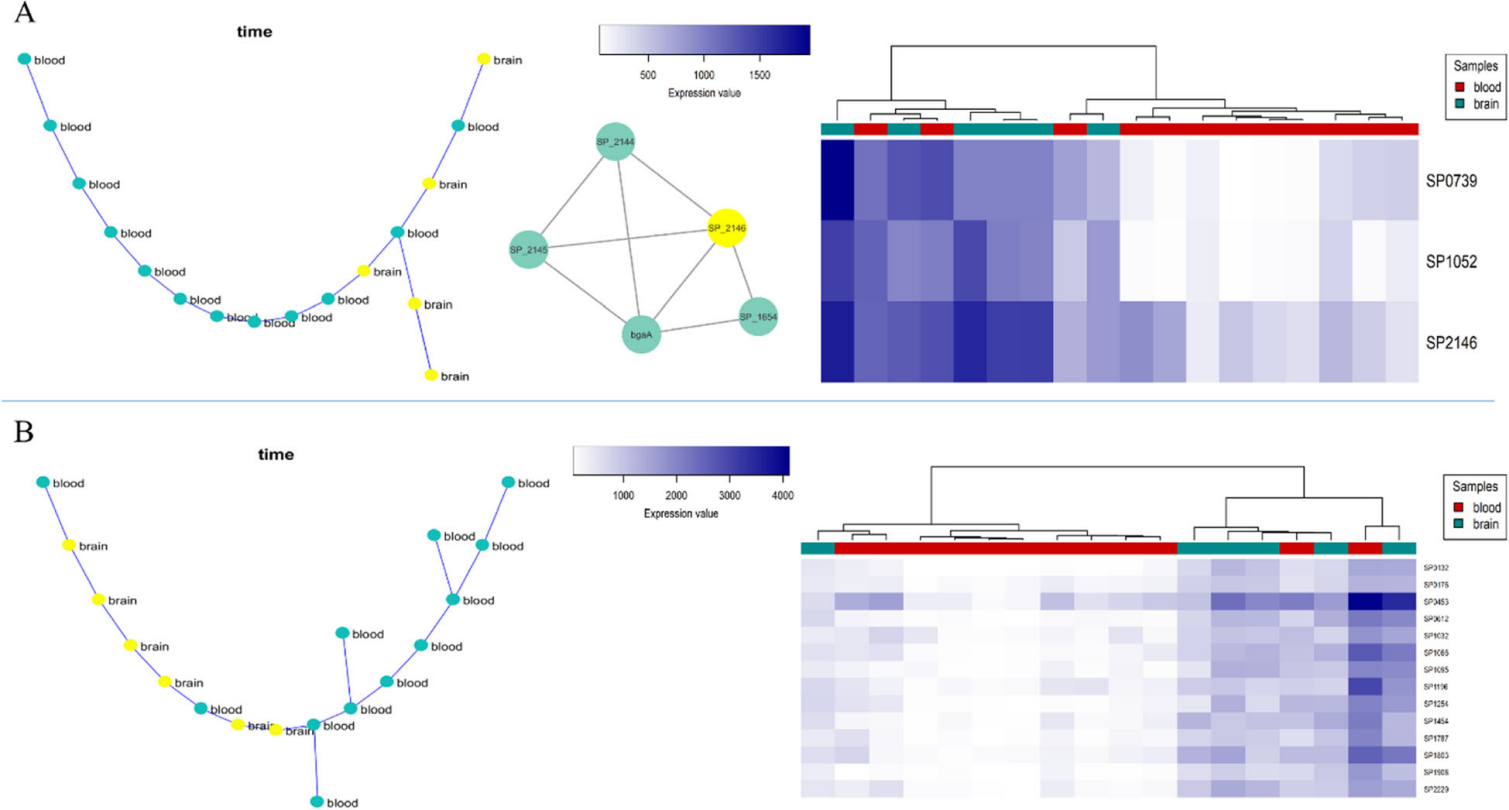
**a** The co-expressed module 130 represents the infection progression in the blood to the brain (Left to right: MST, the genes interact with SP_2146 based on STRING database, hierarchical clustering based on gene expression data). **b** The co-expressed module 87 represents the infection progression in the blood to the brain (Left: MST, Right: hierarchical clustering based on gene expression data)

In blood-brain data, 138 modules were identified, among which, based on MST results, modules 130 and 87 are present mostly co-expressed genes in the infection progression from blood to lung (Fig. 3). Interestingly, although module 130 contained only three genes, the MST and hierarchical clustering results showed that the expression pattern was significantly different between the blood and brain. We could not find any pathway or process for these genes through enrichment analysis, but in the STRING database, the gene SP_2146 interacts with four other genes, including SP_2144, SP_2145, SP_1654, and SP_0648 (*bgaA*) (Fig. 3a). In addition, SP_2146, SP_1654 and SP_0648 are involved in “other glycan degradation” pathway (*p*-value = 3.00e-05). Robb et al. [20] demonstrate that this pathway is required for full virulence in *Streptococcus pneumonia*.

For module 87, no result was obtained from enrichment analysis; however, the gene SP_0176 (ribAB) interacts with three other genes (SP_0177 or ribE, SP_0175 or ribH, and SP_0178 or ribD) in the bacterial interactome. These four genes were significantly enriched to the “Riboflavin metabolism” pathway (*p*-value = 6.82e-06), a critical pathway in pneumococcal infections that its regulatory factors have been previously introduced as novel drug targets [21, 22].

#### Nasopharynx-lung-blood progression

**Fig. 4:**
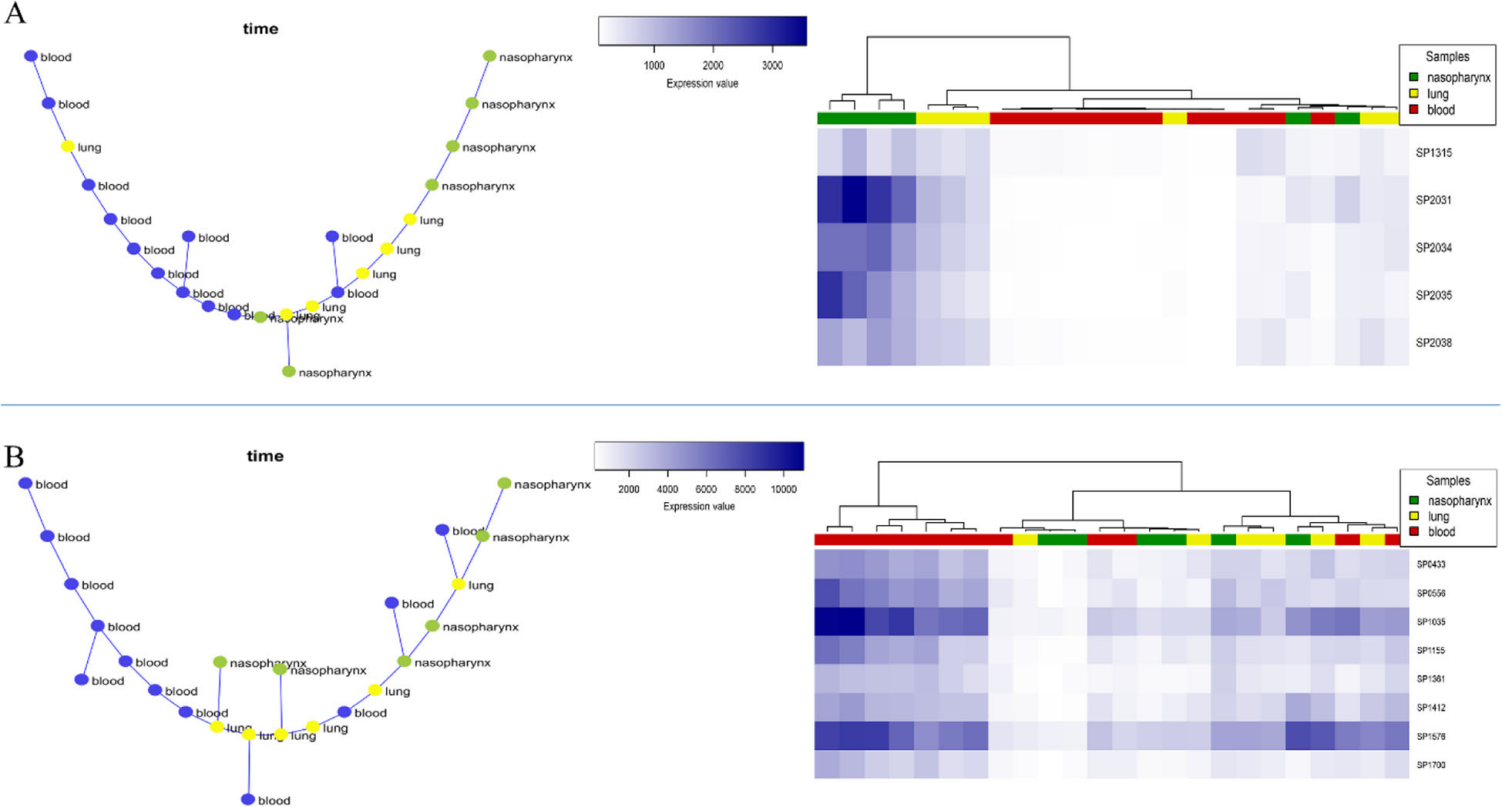
**a** The co-expressed module 95 represents the infection progression from the nasopharynx to lung and blood (Left: MST, Right: hierarchical clustering based on gene expression data). **b** The co-expressed module 103 represents the infection progression from the nasopharynx to lung and blood (Left: MST, Right: hierarchical clustering based on gene expression data)

Given the data derived from the nasopharynx-lung-blood progression, 179 modules were detected by the SPD algorithm, among which modules 95 and 103 had the best clustering results (Fig. 4). As shown in Fig. 4, the gene expression patterns of these modules can cluster the samples. The heatmaps in Fig. 4 shows that as the infection progresses from the nasopharynx to lung and then to blood, the gene expression values are simultaneously decreased in module 95 and increased in module 103. The genes in module 95 and 103 are significantly enriched to “Ascorbate and Aldarate” and “Cysteine and methionine” metabolic pathways, respectively (S2 Table).

#### Lung-blood-brain progression

**Fig. 5:**
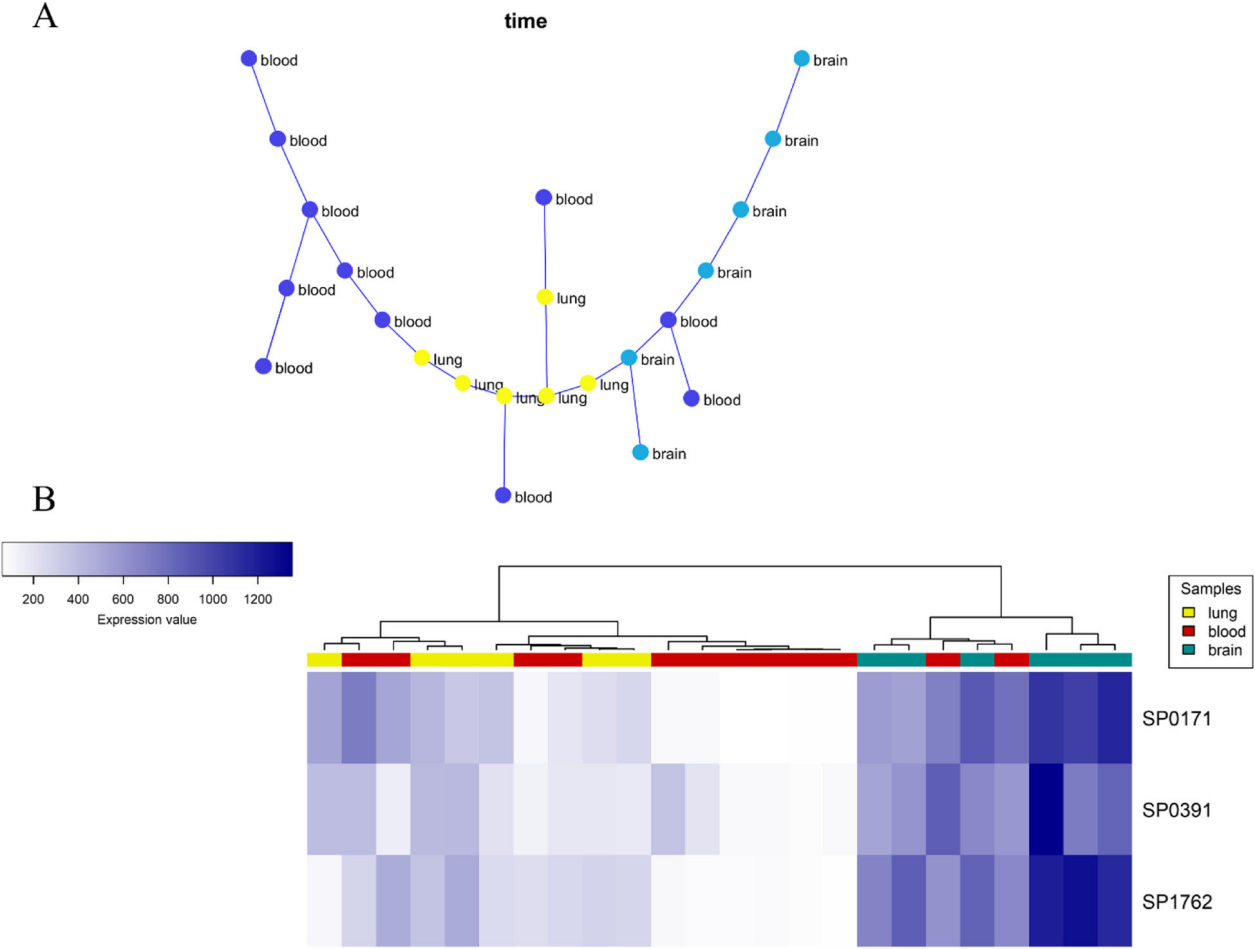
The co-expressed module 30 represents the pneumococcal infection in the lung-blood-brain progression (**a**: MST, **b**: hierarchical clustering based on gene expression data)

Regarding the lung-blood-brain data, 160 modules were detected, in which just one module (module 30) had appropriate results. As shown in Fig. 5, this module contained three genes, including SP_0171, SP_0391 or *CbpF*, and SP_1762.

#### Nasopharynx-lung-blood-brain progression (full progression)

**Fig. 6:**
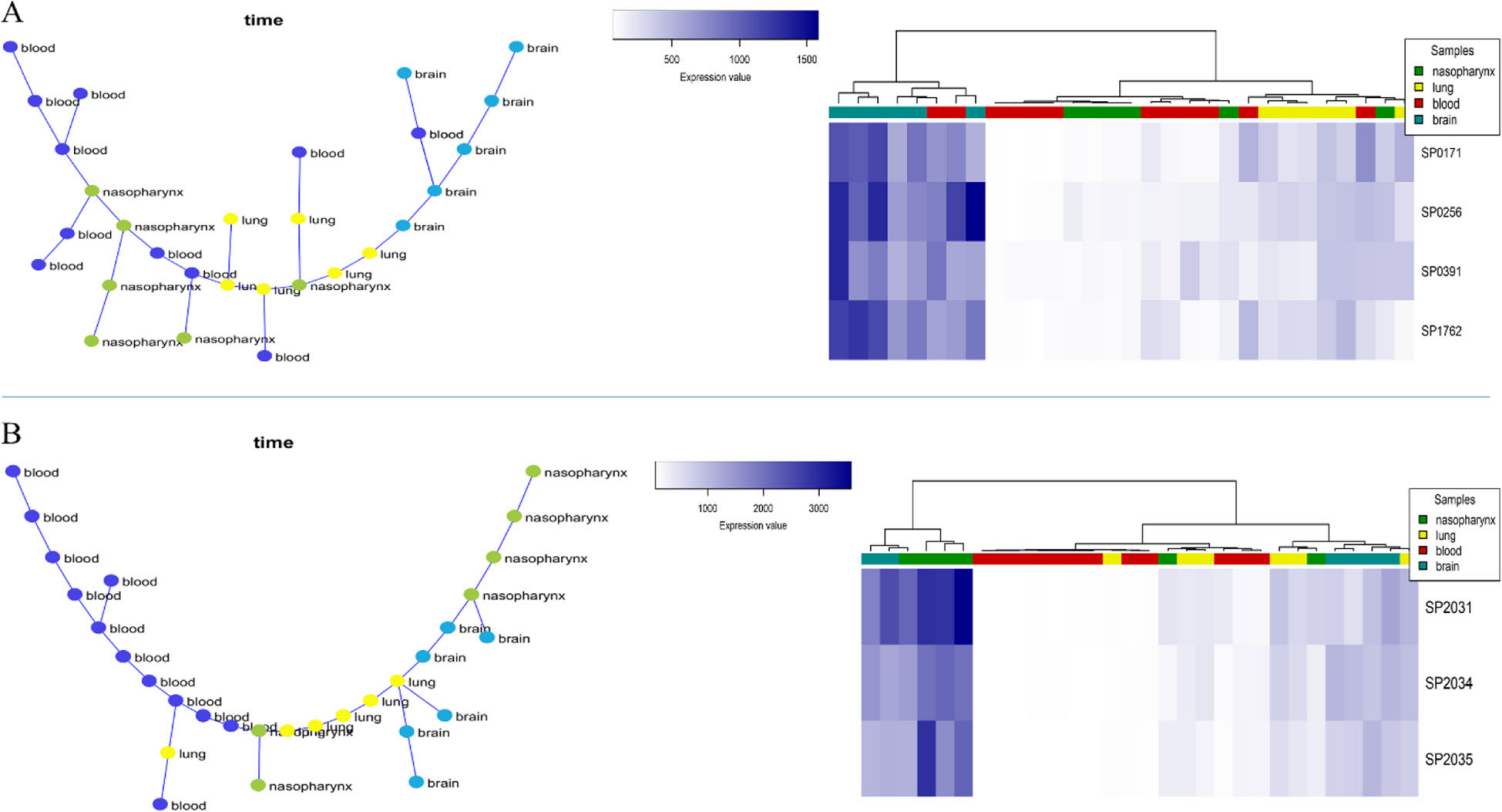
**a** The co-expressed module 34 represents the infection progression in the nasopharynx-lung-blood-brain model (Left: MST, Right: hierarchical clustering based on gene expression data). **b** The co-expressed module 144 represents the infection progression in the nasopharynx to lung, blood, and brain (Left: MST, Right: hierarchical clustering based on gene expression data)

In this section, we considered all samples’ data to find co-expressed modules that their expression patterns were significantly changed throughout all samples. We could identify 169 modules, among which two modules, including modules 34 and 144, had the optimal results (Fig. 6). Module 34 contained four genes, including SP_0171, SP_0256, SP_0391, and SP_1762, three of which are common with module 30 genes detected in the lung-blood-brain progression data. No pathways or processes were found via enrichment analysis for this module. Regarding module 144, three genes, including SP_2031, SP_2034, and SP_2035, were found. As shown in Fig. 6, the module’s MST and hierarchical clustering results indicated a significantly different gene expression pattern compared to all samples. Besides, this module’s genes were significantly enriched to the “Ascorbate and aldarate metabolism” pathway (S2 Table).

### Differentially expressed genes vs. SPD results

**Fig. 7:**
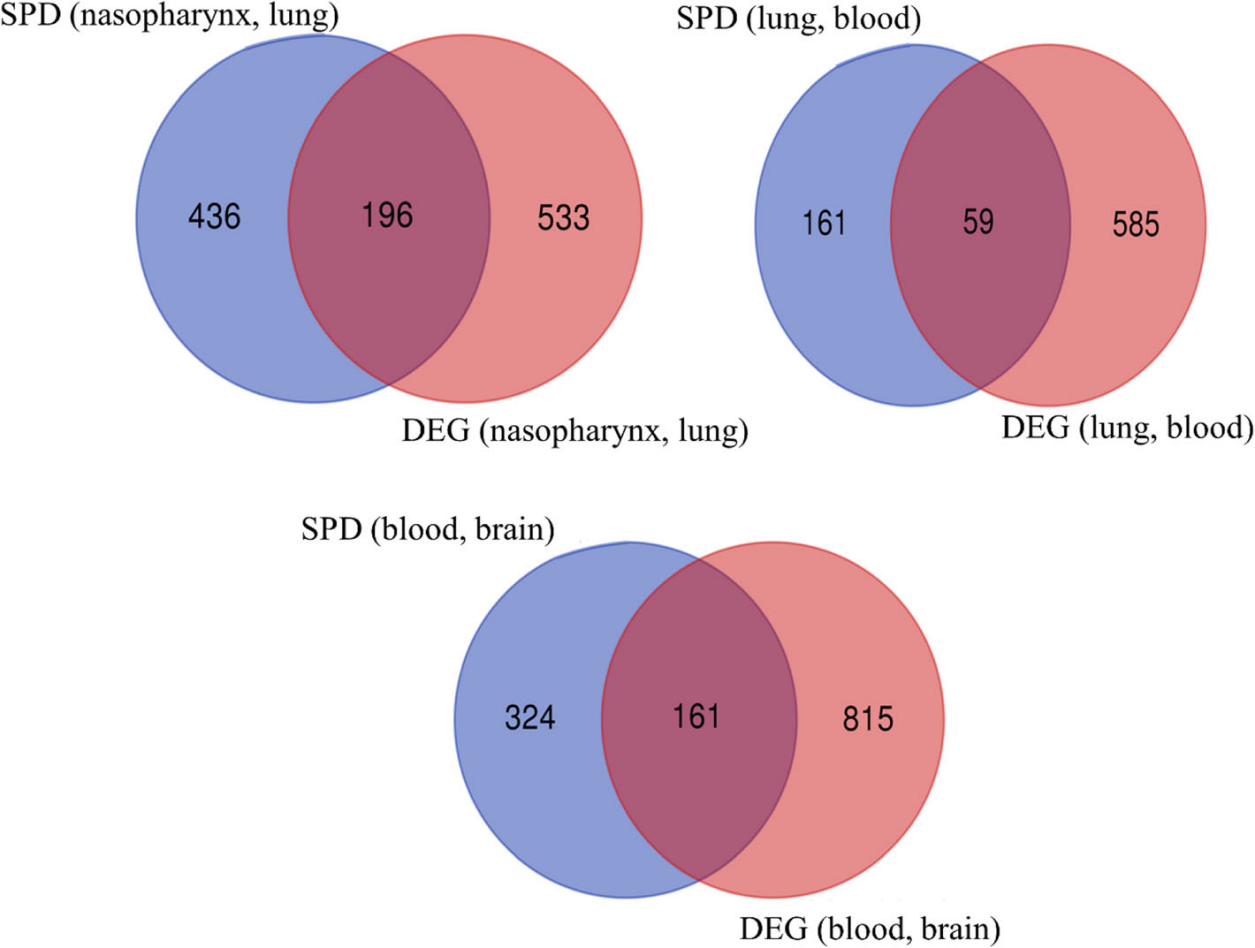
The Venn diagram of the SPD results and DEGs (|logFC| > 0.7 and *p*-value< 0.5)

Considering the infection progression from the nasopharynx to the brain, we compared the differentially expressed genes (DEGs, S2 File) with the genes obtained from SPD analysis when the pneumococci transfer from one sample niche to another one. The number of co-expressed SPD detected genes and DEGs is shown in Table 2. Also, the Venn diagram of the SPD identified genes and DEGs is shown in Fig. 7. As shown in Table 2, the number of DEGs (with both logFC thresholds) when the bacteria move from blood to the brain is higher than in other conditions. This evidence may indicate the high bacterial transcriptome alteration through infection progression from blood to the brain. In contrast, the transcriptome alteration is the least when the infection progressed from the lung to blood (comparing with two other conditions shown in the table). Approximately 10, 15, and 25% of the DEGs are co-expressed and detected by SPD in nasopharynx vs. lung, lung vs. blood, and blood vs. brain conditions.

**Table 2:** The number of DEGs and co-expressed SPD detected genes

Condition	#SPD	#DEG	#Common	logFC
Nasopharynx vs. lung	632	729	196	0.7
		446	105	1
Lung vs. blood	220	644	59	0.7
		327	33	1
Blood vs. brain	485	976	161	0.7
		821	126	1

*P-value< 0.05

Regarding the genes in the SPD modules (S1 file), particularly the modules mentioned earlier, we can find a group of genes that are not classified as DEGs; however, they are critical for the infection process based on previous reports. For instance, as mentioned above, SP_0054 is an early response gene in human lung epithelial cells, while SP_0274 is a crucial gene in pulmonary infection [8, 17]. Table 3 shows some critical infection-related genes detected by the SPD algorithm, while they are not assigned to DEGs.

**Table 3:** The key infection-related genes were not categorized as DEGs but detected by the SPD algorithm

SPD Modules	Gene name	Role in the infection process	Reference (s)
14 (Nasopharynx, lung)	SP_0054	Early response gene in human lung epithelial cells	[8]
	SP_0274	Essential gene in lung infection	[17]
	SP_1460	Involved in iron starvation condition	[16]
	SP_1780	Essential gene in pulmonary infection	[17]
17 (Nasopharynx, lung)	SP_2176	Enriched in Two-component system which controls the virulence and bacterial resistance to oxidative stress	[11, 12]
101(lung, blood)	SP_1923	Vaccine candidate gene	[19]
22 (lung, blood)	SP_0215	Enriched in nitrogen compound metabolism and primary metabolic process which is dysregulated in copper resistance in Streptococcus pneumonia	[18]
	SP_1540		
	SP_1105	Enriched in the metabolic process of nitrogen compounds	[18]
130 (blood, brain)	SP_0739	Up-regulated in response to exposure to penicillin	[23]
	SP_1052	Contributes to virulence in mice	[24]

## Discussion

Extensive transcriptomic changes occur when pneumococci migrate from the nasopharynx into the lung, blood, or brain. Available pneumococcal gene expression studies rely on only DEG genes during bacterial transmission from one body niche to another. According to the systems biology approach, sometimes a gene may not have a significant expression level; however, it could play an important role in the complex system of gene regulation and disease progression. Our study aimed to predict these genes signatures and related alterations during infection progression from the nasopharynx to other niches. Acccordingly, we tried to apply a method on transcriptome data to extract a subset of genes undergoing a spectrum of expression changes between niches. Because co-expressed genes often share common pathways and are involved in common cellular processes [25], appropriate methods should try to extract co-expressed modules instead of single or unrelated altered genes. Therefore, we selected the SPD algorithm as an appropriate method to identify co-expressed modules representing sample progression in transcriptome data. Although other approaches such as differentially co-expressed module identification [26] and Atomic Regulons can be used [27], the SPD algorithm can compare multiple niches simultaneously. Though the feature selection algorithms [28] can detect gene alterations in multiple conditions, they ignore gene-gene interactions and thus were not suitable for our study.

SPD is performed to obtain different sets of genes specific to every niche. For example, SP_0446 and SP_0959 genes were detected in lung-blood migration data. These genes were previously reported as dysregulated genes in the early response in *THP-1 human macrophages* [29]*. In blood-brain* migration *data,* SP_2144 was detected in module 26. This gene, along with two others (SP_2145 and SP_1654), was previously reported as virulence-related genes in *S. pneumonia* [17, 30, 31]. These three genes physically interact with module 130 (detected in blood-brain migration) based on *S. pneumonia interactome.* SPD detects SP_0171 and SP_0391 (cbpF) in module 30 in lung-blood-brain migration data. SP_0171 is a ROK family protein expressed differentially in the early response to THP-1 human macrophage [29]. SP_0391 (*cbpF*) is an important choline-binding protein that was reported previously as a virulence factor in *S. pneumonia* [32–34]. In full progression data, SP_0391 and SP_0256 are two important co-expressed genes that were detected by SPD in module 34. SP_0391 is an important virulence factor of pneumococci [32–34], and SP_0256 is up-regulated in response to penicillin [23]. Furthermore, enrichment analysis revealed some important pathways and processes which may play an important role in pneumococcal infection. For instance, the genes in module 22, detected in lung-blood progression data, significantly enriched to “nitrogen compound metabolic process.” Lui et al. demonstrated that the genes deferentially expressed in children with acute otitis media due to *Streptococcus pneumonia* are significantly enriched in this process [35]. As mentioned in the result section, in nasopharynx-lung-blood migration data, module 95 and 103 are significantly enriched to “Ascorbate and aldarate metabolism” and “Cysteine and methionine metabolism” pathways, respectively. Previous studies demonstrated that ascorbic acid metabolism affects the expression of some critical genes in the pathogenicity of *S. pneumonia* [36]. Also, methionine synthesis has a critical role in bacterial growth and virulence [37]. Identified genes may be applied as antibacterial therapeutic targets and vaccine candidates after more investigations, including determining the cellular location. There are some methodological limitations and problems in this study: 1) Data acquisition; at first, we tried to search for microarray and RNA-Seq data from public datasets, such as GEO and ArrayExpress, using the keyword “Streptococcus pneumonia.” There was a great limitation since the systems biology approach requires a high number of clinical samples. Unfortunately, we found that high-throughput data are scarce for most bacteria, including *S. pneumonia*, and in contrast to many other human-related fields, including cancer studies, the field of bacterial pathogenesis in systems biology is relatively in its infancy. Accordingly, we had to perform our study on currently available data. We finally found only two appropriate pneumococcal transcriptome datasets that could be integrated to increase the data volume. However, regarding the advent of systems biology approaches in medical bacteriology, this field will be definitely developed in the future. In spite of limited studies with a small sample size, the sample size is still critical to achieving precise statistical significance in systems biology. Pooling data from similar studies, if logically permissible, could overcome to some extent the problems. Therefore, it is imperative to conduct larger volume studies and use a high number of samples to generate applicable high-throughput data. Due to the emerging of high-throughput technology, such as RNA-Seq, the data limit will be diminished, and in future studies, machine-learning approaches, such as SPD, could be applied to new appropriate datasets to extract significant results. 2) Another critical issue is that available databases are not exclusively devoted to bacteria, and their search tools are publicly designed, making it difficult to explore bacterial data. We could only search and use available datasets in spite of their limitations. For this reason, we were not capable of interpreting some of the obtained results. Accordingly, developing a comprehensive bacteria-specific database storing transcriptomic (or other bacterial omics) data, along with specially designed bacterial searching tools, is a valuable and essential step in developing and advancing systems biology studies to more in understanding the pathogenesis of infectious diseases. 3) The lack of an appropriate enrichment tool is another challenging issue in our research. STRING was the only relatively suitable enrichment tool; however, it is not specific for bacteria and may cover very poorly pneumococcal genes. Providing powerful and user-friendly enrichment tools for bacterial pathogens is highly needed. 4) The next challenge was to interpret the results to obtain functional information about these genes and their associated biological processes and pathways through databases. Although there are various databases and many identified biological processes for human and mouse genes, it does not cover most bacteria, including pneumococci. Likewise, no annotation on the function or biological processes was available in databases for numerous discovered pneumococcal genes in this study. This greatly affects the enrichment process because we were not able to provide any interpretation for many modules or gene clusters, although the results showed significant issues.

As a note, although given the small genotype differences between various serotypes, it would be better to use data from one serotype alone for the study; however, for some reason, we utilized only two datasets. First, our study is based on identifying bacterial invasion patterns and based on this, and we can approximately categorize invasive serotypes. Second, we needed to investigate the pathogenesis pattern in several ecological niches, from the normal flora in the nasopharynx to the complete pathogenicity (including meningitis and sepsis). Hence, we used only two studies. Third, each of these studies alone had a small sample size, and by pooling them, the sample volume was increased. Consequently, we believe that the lack of sufficient omics data, bacteria-specific databases, and appropriate tools are the main drawbacks of systems biology and computational research to analyze bacterial pathogens, such as Streptococcus pneumonia. In conclusion, this is the first study using the SPD algorithm to assess the transcriptome pattern of pneumococci in different niches, regardless of the expression fold change of genes. Although some of the genes obtained here are entirely unknown, our results show that the expression patterns of these genes are different in different niches, and some of them interact with important well-known genes at the protein level, emphasizing their importance for more closely recognition. It seems that this approach could identify new essential genes involving in various pneumococcal pathogenesis that have been disregarded in the conventional method (fold change expression analysis). This approach could identify important novel genes. This approach is not specific to pneumococci and is applicable if there is adequate and appropriate data, database, and enrichment tools for any other pathogen.

## Methods

### Data preparation

**Fig. 8:**
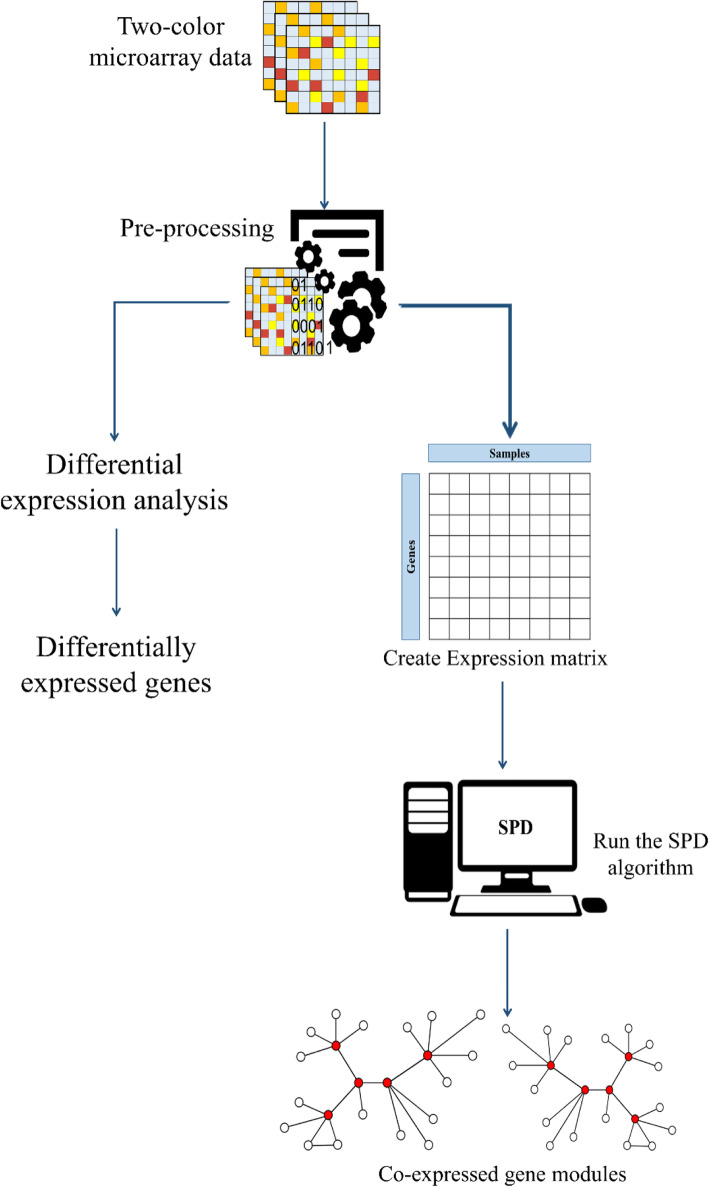
Method workflow

Figure 8 shows our method workflow. The first step of our study is data preparation. The datasets used in this study were downloaded from ArrayExpress database (https://www.ebi.ac.uk/arrayexpress/) with the accession numbers of E-BUGS-130 and E-BUGS-133. The two-color microarray technology had been used to extract the total RNA expression of the bacteria in 48, 72, and 96 h’ time points in a mice intranasal challenge experiment [38, 39]. E-BUGS-130 contains the blood and brain samples, and E-BUGS-133 contains the nasal, blood, and lung samples. The pneumococcal strains included the strain WCH43 (serotype 4) and WCH16 (serotype 6A) isolated from blood. The array’s design is available at the ArrayExpress database with the accession number of A-BUGS-14, and the gene annotations are based on TIGR4 and R6 strains.

### Data processing

After the pre-processing of samples, the background correction and the quantile normalization were applied using the limma R package [40]. The green and red color data were separated in the microarray results to reconstruct an expression matrix containing the expression data of the genes in each experimental condition. Next, the average expression value was replaced for duplicate probes or conditions in each dataset. Finally, the datasets’ expression matrices were combined to reconstruct the final expression matrix (S3 File) for further analysis. In this matrix, the rows represented probes or genes, and the columns represented the experimental conditions (time points and samples).

### Differential gene expression analysis

To compare the SPD results with Differentially Expressed Genes (DEGs), differential gene expression analysis was applied using the limma package [40] in the R programming environment. The genes that are differentially expressed from one niche to another when the infection progressed from nasopharynx to brain were extracted through t-test with *p*-value < 0.05 and |logFC| > 0.7 (|logFC| > 1 was also extracted).

### Module detection and enrichment analysis

To detect co-expressed gene modules representing biological progressions behind time-series microarray data, the SPD algorithm was applied to the expression matrix [6]. Based on each detected module’s expression, a Minimum Spanning Tree (MST, an acyclic graph with minimum total edge weights) was created as columns of the expression matrix for each sample. The weight of each edge denotes the Euclidean distance between two samples, and each tree represents a biological progression in all samples [6]. The pneumococcal infection progression begins from the nasopharynx and can extend to the lung, blood, and brain [41]. Considering the expression data in these niches in multiple time points, we categorized the data into six groups, including 1) [Nasopharynx and lung], 2) [Lung and blood], 3) [Blood and brain], 4) [Nasopharynx, lung, and blood], 5) [Lung, blood, and brain], and 6) finally [Nasopharynx, lung, blood, and brain]. Subsequently, the SPD algorithm was applied to each group with the correlation threshold of 0.95 and the minimum gene cut-off of one to predict the significant modules. After detecting the modules, we compared the MSTs in each group and selected those modules able to separate the body niches based on their expression patterns. These modules were chosen as the best results of the SPD algorithm for further analyses.

### Gene set enrichment analysis and interaction assessment

The gene set enrichment analysis was performed for detected modules using the Comparative GO web tool [42, 43], the STRING database [44] based on KEGG pathways [45], and Gene Ontology Biological Processes [46, 47]. Besides, the STRING database was used for interaction analysis.

### Hierarchical clustering and visualization

Hierarchical clustering and visualization were performed in the R programming environment with the Euclidean distance and Ward.D2 method [48]. The Venn diagrams were drawn with an online tool available at http://bioinformatics.psb.ugent.be/webtools/Venn/. Also, network visualization was performed via Cytoscape software [49].

## Conclusions

In conclusion, this is the first study using the SPD algorithm to assess the transcriptome pattern of pneumococci in different niches, regardless of the expression fold change of genes. Although some of the genes obtained here are entirely unknown, our results show that the expression patterns of these genes are different in different niches, and some of them interact with well-known essential genes at the protein level, emphasizing their importance for more closely recognition. It seems that this approach could identify new essential genes involving in various pneumococcal pathogenesis that have been disregarded in the conventional method (fold change expression analysis). This approach can identify significant novel genes not only in pneumococci but also in other pathogens in the case of the availability of adequate and appropriate data, databases, and enrichment tools.

## Supplementary Information

**Additional file 1:.** SPD genes. All genes were detected by the SPD algorithm.

**Additional file 2:.** DEGs. Differentially expressed genes.

**Additional file 3:.** Expression matrix. The expression matrix was extracted from microarray samples.

**Additional file 4: Table S1.** Genes in the top modules. Co-expressed genes in the best modules extracted from the data by the SPD algorithm.

**Additional file 5: Table S2.** Enrichment Analysis. Biological process and pathway enrichment analysis results for the best modules obtained using the SPD algorithm.

## Data Availability

The datasets are available in the ArrayExpress database with the accession number of E-BUGS-130 and E-BUGS-133. The SPD algorithm source code and user guide are available at http://pengqiu.gatech.edu/software/SPD/index.html. Other source codes and materials are available upon request.

## Abbreviations

SPD: Sample Progression Discovery
COPD: Chronic pulmonary obstructive disease
MST: Minimum spanning tree
DEGs: Differentially expressed genes
IMP: Inosine monophosphate
CAMP: Cationic antimicrobial peptide
