# Supplementary material for: The identification of co-expressed gene modules in Streptococcus pneumonia from colonization to infection to predict novel potential virulence genes
Source: BMC Microbiol. 2020 Dec 17;20:376. doi: 10.1186/s12866-020-02059-0 (PMC7745498; doi:10.1186/s12866-020-02059-0)
Supplement: Supplementary file 4 — Additional file 4: Table S1. Genes in the top modules. Co-expressed genes in the best modules extracted from the data by the SPD algorithm. [file 12866_2020_2059_MOESM4_ESM.docx]

**Table S1.** Co-expressed genes in the best modules extracted from the data by the SPD algorithm.

| Sample | Module | Gene | Annotation |
| --- | --- | --- | --- |
| Nasopharynx and lung | 14 | purH | bifunctional phosphoribosylaminoimidazolecarboxamide formyltransferase/IMP cyclohydrolase |
|  |  | purK | phosphoribosylaminoimidazole carboxylase ATPase subunit |
|  |  | nrdD | anaerobic ribonucleoside triphosphate reductase |
|  |  | SP_0204 | acetyltransferase |
|  |  | nrdG | anaerobic ribonucleoside-triphosphate reductase activating protein |
|  |  | polC | DNA polymerase III/PolC |
|  |  | SP_0804 | 4-methyl-5(b-hydroxyethyl)-thiazole monophosphate biosynthesis protein |
|  |  | SP_0864 | hypothetical protein |
|  |  | SP_1249 | guanosine 5'-monophosphate oxidoreductase |
|  |  | SP_1460 | amino acid ABC transporter ATP-binding protein |
|  |  | SP_1780 | oligoendopeptidase F |
|  |  | SP_1880 | deoxyribonucleotide triphosphate pyrophosphatase/unknown domain fusion protein |
|  |  | spxA | transcriptional regulator Spx |
|  | 71 | SP_0055 | hypothetical protein |
|  |  | SP_0819 | IS630-Spn1, transposase Orf2 |
|  |  | SP_0902 | hypothetical protein |
|  |  | SP_1245 | Cof family protein |
|  |  | SP_1247 | hypothetical protein |
|  |  | SP_1779 | hypothetical protein |
|  |  | SP_2014 | IS630-Spn1, transposase Orf2 |
|  |  | SP_2173 | dltD protein |
|  |  | SP_2175 | dltB protein |
|  |  | SP_2176 | D-alanine--poly(phosphoribitol) ligase subunit 1 |
| Lung and blood | 22 | rpsC | 30S ribosomal protein S3 |
|  |  | rplP | 50S ribosomal protein L16 |
|  |  | rpsQ | 30S ribosomal protein S17 |
|  |  | rplR | 50S ribosomal protein L18 |
|  |  | rpsE | 30S ribosomal protein S5 |
|  |  | rplU | 50S ribosomal protein L21 |
|  |  | SP_1540 | single-stranded DNA-binding protein |
|  | 101 | ilvN | acetolactate synthase 3 regulatory subunit |
|  |  | infC | translation initiation factor IF-3 |
|  |  | SP_1073 | RNA polymerase sigma factor RpoD |
|  |  | SP_1665 | hypothetical protein |
|  |  | SP_1737 | DNA-directed RNA polymerase subunit omega |
|  |  | SP_1891 | oligopeptide ABC transporter oligopeptide-binding protein AmiA |
|  |  |  | pneumolysin |
|  |  | SP_1942 | transcriptional regulator |
|  |  | SP_2015 | IS630-Spn1, transposase Orf1 |
| Blood and brain | 130 | SP_0739 | MerR family transcriptional regulator |
|  |  | SP_1052 | hypothetical protein |
|  |  | SP_2146 | hypothetical protein |
|  | 87 | SP_0132 | pseudo |
|  |  | ribAB | 3,4-dihydroxy-2-butanone 4-phosphate synthase |
|  |  | SP_0453 | amino acid ABC transporter amino acid-binding protein/permease |
|  |  | SP_0612 | hypothetical protein |
|  |  | SP_1032 | iron-compound ABC transporter iron compound-binding protein |
|  |  | SP_1066 | pseudo |
|  |  | SP_1095 | ribose-phosphate pyrophosphokinase |
|  |  | SP_1196 | pseudo |
|  |  | SP_1254 | hypothetical protein |
|  |  | SP_1454 | hypothetical protein |
|  |  | SP_1787 | hypothetical protein |
|  |  | SP_1803 | hypothetical protein |
|  |  | SP_1908 | single-stranded DNA-binding protein |
|  |  | SP_2229 | tryptophanyl-tRNA synthetase |
| Nasopharynx, lung, and blood | 95 | SP_1315 | V-type ATP synthase subunit D |
|  |  | SP_2031 | L-ascorbate 6-phosphate lactonase |
|  |  | SP_2034 | L-xylulose 5-phosphate 3-epimerase |
|  |  | ulaD | 3-keto-L-gulonate-6-phosphate decarboxylase |
|  | 103 | nusB | transcription antitermination protein NusB |
|  |  | infB | translation initiation factor IF-2 |
|  |  | SP_1035 | iron-compound ABC transporter ATP-binding protein |
|  |  | SP_1361 | homoserine dehydrogenase |
|  |  | SP_1412 | prolipoprotein diacylglyceryl transferase |
|  |  | SP_1576 | homoserine O-succinyltransferase |
|  |  | SP_1700 | phospho-2-dehydro-3-deoxyheptonate aldolase |
| Lung, blood, and brain | 30 | SP_0171 | ROK family protein |
|  |  | CbpF | choline binding protein F |
|  |  | SP_1762 | hypothetical protein |
| Nasopharynx, lung, blood, and brain | 34 | SP_0171 | ROK family protein |
|  |  | SP_0256 | acetyltransferase |
|  |  | SP_0391 | choline binding protein F |
|  |  | SP_1762 | hypothetical protein |
|  | 144 | SP_2031 | L-ascorbate 6-phosphate lactonase |
|  |  | SP_2034 | L-xylulose 5-phosphate 3-epimerase |
|  |  | ulaD | 3-keto-L-gulonate-6-phosphate decarboxylase |
