# Supplementary material for: The identification of co-expressed gene modules in Streptococcus pneumonia from colonization to infection to predict novel potential virulence genes
Source: BMC Microbiol. 2020 Dec 17;20:376. doi: 10.1186/s12866-020-02059-0 (PMC7745498; doi:10.1186/s12866-020-02059-0)
Supplement: Supplementary file 5 — Additional file 5: Table S2. Enrichment Analysis. Biological process and pathway enrichment analysis results for the best modules obtained using the SPD algorithm. [file 12866_2020_2059_MOESM5_ESM.docx]

**Table S2.** Biological process and pathway enrichment analysis results for the best modules obtained uning the SPD algorithm.

| Samples | Module | Term description | Source | Matching genes in the module | P-value |
| --- | --- | --- | --- | --- | --- |
| Nasopharynx and lung | 14 | 'de novo' IMP biosynthetic process | GO biological process | SP_0054, SP_0050 | 0.0008 |
|  |  | Purine metabolism | KEGG | SP_0050, SP_0054, SP_0202, SP_0274, SP_1249, SP_1880 | 3.29e-06 |
|  | 71 | Cationic antimicrobial peptide (CAMP) resistance | KEGG | SP_2173, SP_2175, SP_2176 | 1.84e-05 |
|  |  | Two-component system | KEGG | SP_2173, SP_2175, SP_2176 | 0.00079 |
| Lung and blood | 22 | Ribosome | KEGG pathway | SP_0215, SP_0216, SP_0218, SP_0226, SP_0227, SP_1105, SP_1540 | 1.17e-08 |
|  |  | cellular macromolecule biosynthetic process | GO biological process | SP_0215, SP_0216, SP_0218, SP_0226, SP_0227, SP_1105 | 4.44e-07 |
|  |  | translation | GO biological process | SP_0215, SP_0216, SP_0218, SP_0226, SP_0227, SP_1105 | 7.03e-07 |
|  |  | peptide metabolic process | GO biological process | SP_0215, SP_0216,S P_0218, SP_0226, SP_0227, SP_1105, SP_1540 | 7.03e-07 |
|  |  | cellular nitrogen compound metabolic process | GO biological process | SP_0215, SP_0216, SP_0218, SP_0226, SP_0227, SP_1105, SP_1540 | 1.93e-06 |
|  |  | primary metabolic process | GO biological process | SP_0216, SP_1540 | 9.18e-06 |
|  |  | response to stimulus | GO biological process | SP_0215, SP_0216, SP_0218, SP_0226, SP_0227, SP_1105, SP_1540 | 0.0192 |
| Nasopharynx, lung, and blood | 95 | Ascorbate and aldarate metabolism | KEGG pathway | SP_2031, SP_2034, SP_2035, SP_2038 | 2.50e-08 |
|  |  | Microbial metabolism in diverse environments | KEGG pathway | SP_2031, SP_2034, SP_2035, SP_2038 | 6.11e-05 |
|  |  | Pentose and glucuronate interconversions | KEGG pathway | SP_2034, SP_2035 | 0.00026 |
|  |  | Metabolic pathways | KEGG pathway | SP_1315, SP_2031, SP_2034, SP_2035, SP_2038 | 0.00026 |
|  | 103 | Cysteine and methionine metabolism | KEGG pathway | SP_1361, SP_1576 | 0.0267 |
|  |  | Biosynthesis of amino acids | KEGG pathway | SP_1361, SP_1576, SP_1700 | 0.0267 |
|  |  | Biosynthesis of secondary metabolites | KEGG pathway | SP_1361, SP_1576, SP_1700 | 0.0486 |
| Nasopharynx, lung, blood, and brain | 144 | Ascorbate and aldarate metabolism | KEGG pathway | SP_2031, SP_2034, SP_2035 | 5.49e-07 |
|  |  | Pentose and glucuronate interconversions | KEGG pathway | SP_2034, SP_2035 | 9.52e-05 |
|  |  | Microbial metabolism in diverse environments | KEGG pathway | SP_2031, SP_2034, SP_2035 | 0.00012 |
|  |  | Metabolic pathways | KEGG pathway | SP_2031, SP_2034, SP_2035 | 0.0046 |
